# Additional records of *Neopsittaconirmus* lice (Insecta: Phthiraptera: Ischnocera) parasitizing captive parrots

**DOI:** 10.1590/S1984-29612025004

**Published:** 2025-02-03

**Authors:** Yoshika Oniki-Willis, Oldrich Sychra, Ricardo Luis Palma

**Affiliations:** 1 Instituto de Estudos da Natureza, Organização Não Governamental, Rio Claro, SP, Brasil; 2 Department of Biology and Wildlife Diseases, Faculty of Veterinary Hygiene and Ecology, University of Veterinary Sciences Brno, Brno, Czechia; 3 Museum of New Zealand, Te Papa Tongarewa, Wellington, New Zealand

**Keywords:** Ectoparasites, budgerigar, Melopsittacus undulatus, cockatiel, Nymphicus hollandicus, Ectoparasitas, periquito australiano, Melopsittacus undulatus, calopsita, Nymphicus hollandicus

## Abstract

Species of the chewing louse genus *Neopsittaconirmus* Conci, 1942 are host-specific parasites on Old World and Australasian parrots (Psittaciformes), infesting both wild and captive birds. Despite veterinarian practices that attempt to eliminate parasites from captive hosts, two species, *Neopsittaconirmus gracilis* Guimarães, 1974 and *Neopsittaconirmus vendulae* Sychra, 2006, frequently infest captive birds, not only their regular natural hosts, but also other species that are not naturally parasitized. Here we report and discuss additional records of these two species of lice from captive parrots in Brazil, Australia, England, Réunion and the United States of America.

## Resumo

Espécies de piolho mastigador do gênero *Neopsittaconirmus* Conci, 1942 são parasitas específicos de papagaios do Velho Mundo e da Australásia (Psittaciformes), infestando aves selvagens e mantidas em cativeiro. Apesar das práticas veterinárias que tentam eliminar parasitas dos hospedeiros em cativeiro, duas espécies, *Neopsittaconirmus gracilis* Guimarães, 1974 e *Neopsittaconirmus vendulae* Sychra, 2006, infestam frequentemente aves em cativeiro, não apenas os seus hospedeiros naturais regulares, mas também outras espécies que naturalmente não são parasitadas. Aqui foram relatados e discutidos registros adicionais destas duas espécies de piolhos de psitacídeos em cativeiro no Brasil, Austrália, Inglaterra, Reunião e Estados Unidos da América.

Lice are permanent ectoparasites of birds and mammals (Marshall, 1981). They are often host-specific (Price et al., 2003) but may transfer and establish on novel hosts naturally or accidentally by human activities. In natural scenarios, lice may transfer to “foreign” hosts by accidental contact, e.g., during roosting, feeding, sharing dusting bowls, or by phoresy (Lee et al., 2022). This natural dispersal is known as “straggling”. Accidental transfers or contaminations of lice among live hosts of different species by human agencies may occur where live animals are handled, for example, in zoological gardens, pet shops, aviaries, or rehabilitation centers (Galloway, 2023).

The genus *Neopsittaconirmus* Conci, 1942 includes over 30 species of lice mainly parasitic on members of the bird order Psittaciformes – parrots, cockatoos, lorikeets, lovebirds, budgerigars, cockatiels, etc. – from the Old World and Australasia. One exception is *Neopsittaconirmus inexpectatus* Guimarães, 1974 which parasitizes the African pygmy falcon, *Polihierax semitorquatus* (Smith, 1836), Falconiformes (Price et al., 2003: 200). Another possible exception is *Neopsittaconirmus trinoton* (Piaget, 1890), recorded from *Ara macao* (Linnaeus, 1758), a bird from the neotropics; however, Guimarães (1974) referred to it as a *species incertae sedis*, with its host association being most likely erroneous. In natural conditions, all known species of *Neopsittaconirmus* are host- specific, but some species infest captive birds of both their natural regular host species and accidental hosts.

Naz et al. (2024) summarised records of three species of *Neopsittaconirmus* from captive parrots held in several countries in Europe, Asia and the Americas. In Brazil, Gois et al. (2022) reported *Neopsittaconirmus* sp. on captive cockatiels (*Nymphicus hollandicus* (Kerr, 1793), Cacatuidae, Psittaciformes) from the northeastern state of Piauí, a record later identified as *Neopsittaconirmus gracilis* Guimarães, 1974 by Naz et al. (2024: 5). Also in Brazil, Neiva & Martins (2021) reported *Neopsittaconirmus* sp. on captive cockatiels from the state of Espírito Santo, which were identified as *Neopsittaconirmus vendulae* Sychra, 2006 by Naz et al*.* (2024: 5).

Here we report and discuss additional historical records of *N. gracilis* and *N. vendulae* collected from captive hosts, found in the collections of the Museu de Zoologia da Universidade de São Paulo (MZUSP, Brazil) and the Museum of New Zealand Te Papa Tongarewa (MONZ, New Zealand).

## *Neopsittaconirmusgracilis* Guimarães, 1974

The first author (Y.O.-W.) obtained and examined a dead budgerigar, *Melopsittacus undulatus* (Shaw, 1805) (Psittaculidae: Psittaciformes), from the local zoological garden in Barão Geraldo, Campinas, State of São Paulo, Brazil. The origin of this budgerigar is unknown, but we believe it was almost certainly bred in captivity. Five males and three females of *Neopsittaconirmus* identified as *N*. *gracilis* were found on this bird. These eight lice are slide-mounted on four slides with additional data: Y.O.-Willis, 10 March 1981, N^os^ 887–890, and deposited in MZUSP.

Guimarães (1974: 169) designated the African yellow-collared lovebird – *Agapornis personatus* Reichenow, 1887 (Psittaculidae, Psittaciformes) – as the type host of *N. gracilis*, based on only three specimens, a male and two females from the Meinertzhagen Collection held in the Natural History Museum, London. Presumably, that lovebird is also a regular and natural host of *N. gracilis*, but many lice held in the Meinertzhagen Collection have been shown to be the result of contaminations (e.g., Palma & Pilgrim, 1984; Palma, 1994). Furthermore, *N. gracilis* has also been recorded several times on budgerigars in captivity and in Australia, where budgerigars naturally occur (Sychra, 2005; Naz et al., 2024: table 4, fig. 21). It is likely that *Agapornis personatus* is the natural regular host of *N. gracilis*, and that records of this louse on budgerigars are the result of contaminations between these two parrots in captivity. However, considering the scarcity of records of *N. gracilis* from both host species in the wild, whether budgerigars are also regular and natural hosts of *N. gracilis* remains unanswered. Further samples of *Neopsittaconirmus* from birds caught in the wild are needed to resolve that question, and confirm the lovebird as a regular and natural host of *N. gracilis*.

## *Neopsittaconirmusvendulae* Sychra, 2006

The first author (Y.O.-W.) collected 16 specimens (8 males, 7 females and 1 nymph) of *Neopsittaconirmus* from a captive cockatiel held in Casa Xororó, Rio Claro, São Paulo, Brazil. This sample – with additional data: N^o^ 1700, Y.O. Willis, 21 July 1985 – was examined by L.R. Guimarães, who stated: “These lice belong to a new species very similar, if not identical to samples (two slides with 3 females each) from *Calopsitta* [= *Nymphicus hollandicus*] that I received from the Zoo of St. Denis, Île de la Réunion. However, they show slight differences in size and chaetotaxy”. The samples from Île de la Réunion were sent to L.R. Guimarães by Dr Nicolas Barré, and they are deposited at MZUSP. We have identified all the lice mentioned above as *Neopsittaconirmus vendulae*, originally described from material collected from a dead, captive cockatiel in Czechia (Sychra, 2006) and later reported from several other countries (Naz et al., 2024: table 4, fig. 21).

Two additional samples of *N. vendulae* from captive cockatiels, held in MONZ and identified by the third author (R.L.P.), have not yet been reported in the literature. They are: One male collected by P.R. Kettle in November 1975 at the Wickham Laboratory, Hampshire, England (MONZ slide AI.020631), and one female collected by C.C. Bullmore in 1983 in California, U.S.A. (MONZ slide AI.017071), which was incorrectly identified by Bullmore (1983) as *Pectinopygus* sp. An additional female of *N. vendulae* was collected from a cockatiel by B. Genn on 10 October 1964 in Brisbane, Queensland, Australia (MONZ slide AI.017070), but it is not known if the host was wild or a captive bird.
